# Label-Free Detection of Biochemical Changes during Cortical Organoid Maturation via Raman Spectroscopy and Machine Learning

**DOI:** 10.1021/acs.analchem.4c05661

**Published:** 2025-02-24

**Authors:** Giulia Bruno, Michal Lipinski, Koseki J. Kobayashi-Kirschvink, Christian Tentellino, Peter T. C. So, Jeon Woong Kang, Francesco De Angelis

**Affiliations:** † Istituto Italiano di Tecnologia, via Morego 30, 16163 Genova, Italy; ‡ G. R. Harrison Spectroscopy Laboratory, Massachusetts Institute of Technology, Cambridge, Massachusetts 02139, United States; § Broad Institute of MIT and Harvard, 415 Main Street, Cambridge, Massachusetts 02142, United States

## Abstract

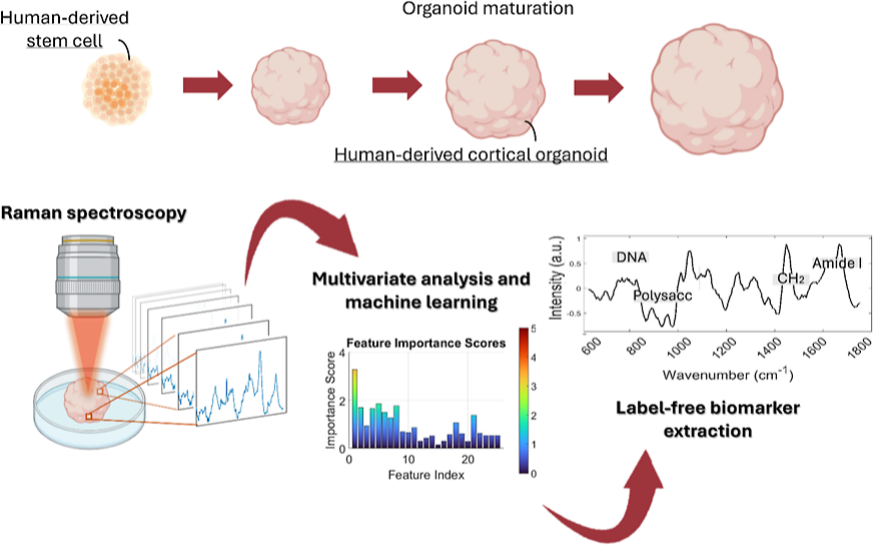

Human cerebral organoids have become valuable tools in neurodevelopment research, holding promise for investigating neurological diseases and reducing drug development costs. However, clinical translation and large-scale production of brain organoids face challenges due to invasive methodologies such as immunohistochemistry and omics that are traditionally used for their investigation. These hinder real-time monitoring of organoids and highlight the need for a nondestructive approach to promote resource-efficient production and standardization and enable dynamic studies for drug testing and developmental monitoring. Here, we propose a label-free methodology utilizing Raman spectroscopy (RS) and machine learning to discern cortical organoid maturation stages and to observe their biochemical variations. We validated the method’s robustness by analyzing both pluripotent stem cell-derived organoids and embryonic stem cell-derived organoids, revealing also significant biochemical variability between the two. This finding paves the way for the use of RS for longitudinal studies to observe dynamic changes in brain organoids, offering a promising tool for advancing our understanding of brain development and accelerating drug discovery.

In recent years, human-derived cerebral organoids have emerged as a focal point of research, holding huge potential for advancing our comprehension of neurodevelopment, neurological disorders, and neurodegenerative diseases. Moreover, organoids offer not only to accelerate drug discovery and development but also to potentially reduce the exorbitant expenses associated with clinical trials.^1−6^ These three-dimensional cellular ensembles possess the ability to replicate the structural and functional characteristics of the human brain and serve as an excellent model for exploring the intricacies of brain development and function. Over the past few years, our team has refined organoid culture methods, yielding a diverse yet consistent cell type representation of the human cerebral cortex. Cultivated from human pluripotent stem cells (hiPSC) and embryonic stem cells (hESC), our organoid model provides invaluable insights into the complex processes governing cortical development.^7^ Researchers have extensively investigated the movement, migration, and differentiation of cells during brain organoid development;^8,9^ however, despite their importance for modeling early stage brain development, the resource-intensive and time-consuming nature of organoid production can pose an obstacle for clinical translation.^10,11^ The fact that destructive techniques like immunohistochemistry or single-cell RNA-seq (scRNA-seq) used to investigate the organoid model are still a gold standard for the field slowed down further improvements in the clinical domain. Immunohistochemistry (IHC) and immunofluorescence are vital for visualizing proteins and cellular markers in organoids, revealing their composition and distribution;^12^ however, the use of fluorescent tags can be perturbative as it can alter cellular interactions and processes,^13^ limiting their applicability, especially in pharmaceutical contexts. On the other hand, scRNA-seq^14^ and other approaches such as proteomics and metabolomics provide insights into cellular diversity, gene expression, and holistic views of molecular landscapes.^15^ However, these methods are destructive, resource-intensive, and generate complex data sets that pose challenges for univocal interpretation.^16^ Unfortunately, these methodological limitations not only deplete the limited supply of organoids but also hinder tracking organoid development over time, which is essential for understanding their dynamic changes and responses to experimental conditions.

To overcome these challenges, it is imperative to develop a label-free, noninvasive technique capable of assessing the physiological status of organoids while complementing other methods to reduce the required organoids usage. Raman spectroscopy (RS) has recently come to the forefront as a promising approach to address these challenges,^17^ offering the unique potential to provide valuable insights into the maturation stages and biochemical variations of organoids and spheroids^18^ without affecting their viability or disturbing their ongoing development. Raman microscopy offers a unique opportunity for observing live cells and tissues, as it identifies the vibrational energy levels of various molecules, including nucleic acids, proteins, and metabolites.^19^ This technique is both label-free and noninvasive, enabling the acquisition of detailed molecular information at subcellular resolution, effectively creating unique molecular profiles of cells. Unlike other noninvasive techniques such as multiphoton imaging,^20^ which is highly sensitive to structural proteins like collagen but lacks biochemical specificity, or optical coherence tomography (OCT),^21^ which excels in imaging tissue architecture but provides limited molecular information, Raman microscopy uniquely combines spatial resolution with detailed molecular profiling, making it an unparalleled tool for noninvasive monitoring of biological systems. The integration of multivariate analysis and machine learning (ML) methodologies into RS^22^ has revolutionized data analysis, releasing it from operator bias and emphasizing statistical relevance. This advancement greatly facilitates the extraction of the specific Raman features from highly variable samples and complex spectra.^23^ Aligned with this trajectory, Tubbesing et al. have demonstrated that Raman spectral fingerprints can be used to noninvasively distinguish among organoid phenotypes in fixed and living salivary gland organoids.^24^ Meanwhile, Pettinato et al. have developed a protocol involving confocal light absorption and scattering spectroscopic microscopy and RS to evaluate chromatin level and biochemical composition of liver transplantable organoid.^25^ In recent years, the Stevens’ group have developed a quantitative RS approach, advancing the capabilities for quantitative chemometric analysis and biomolecular imaging within three-dimensional tissues.^26^ Their recent work (2023), LaLone et al.,^27^ demonstrated the ability to successfully chemotype the majority of biomolecules in three-dimensional liver organoids using RS.

Herein, we demonstrate the capability of Raman spectroscopy in dynamically studying cortical organoids with a specific focus on discrimination between living organoids at different maturation stages. Through the use of random forest (RF) algorithm,^28^ we identify variations in biomarkers such as glycogen, nucleic acids, and lipids, enabling the distinction between these stages. This marks the capability to discern biomolecular changes within the cortical organoids’ maturation process. Our specific approach relies on the analysis of the dynamics of the outermost layer containing most metabolically healthy cells^16,29^ to discern and identify the maturation stage of the organoid. We accomplished this by scanning the external layer of the organoid using a minimal number of fields of view (FOV) rather than a comprehensive scan of the entire organoid. This approach not only increases efficiency but also enables high-resolution imaging, laying the foundation for the assessment of the spatial distribution of biomarkers within the sample. We emphasize the advantages of using RS alongside machine learning for noninvasive assessments of brain organoid growth, thus enabling longitudinal studies and dynamic investigations within the latter. It also underscores the value of RS as a complementary technique to enhance other analytical methods like single-cell sequencing and other omics approaches, contributing to a deeper understanding of brain development and expanding opportunities for drug testing.

## Experimental Section

### Stem Cell Culture and Cortical Organoid Generation

Human male iPSC line PGP1 (Personal Genome Project 1; also called GM23338) was obtained from the laboratory of G. Church. Human male ESC line H1 (also called WA01) was purchased from WiCell. The authentication and growing conditions of both lines were thoroughly described in a previous publication (Uzquiano et al.^16^). Similarly, the cortical organoid differentiation has been described before in Velasco et al.^30,31^

### High-Throughput Multimodal Raman Microscope

In response to the absence of available commercial systems, an automated high-throughput multimodal microscope was designed and extensively discussed in.^17^ To summarize, galvo mirror-based point scanning and stage scanning were used to capture each FOV and many separate FOVs in order to achieve a high-throughput Raman measurement. The acquisition is automated via a MATLAB (2020b) script that interfaces with Micromanager, a DAQ board, and a Raman scattering detector (Princeton Instruments, PIXIS 100BR eXcelon). An IX83 fluorescence microscope body was combined with a 785 nm Raman excitation laser linked to the backport, where the excitation was deflected to the sample via an Olympus UPLSAPO 60X NA 1.2 water immersion objective via a short-pass filter. Backscattered light was collimated via the same objective and captured using a 50 m core multimode fiber before being delivered to the spectrograph and detector (HoloSpec f/1.8i 785 nm model). The bright-field channels were captured using a 270 Hamamatsu Photonics Orca Flash 4.0 v2 sCMOS camera. A 785 nm Ti:Sapphire laser cavity connected to a 532 nm pump laser operating at 4.7 W served as the laser source. The laser intensity at the sample plane was 180 mW, and the exposure period for each point in the Raman experiment was 400 ms. Each FOV was 50 × 50 pixels in size, with a pitch of 1.5 μm. The acquisition of Raman hyperspectral images took around 16 min per FOV. For each organoid, 6 FOVs were chosen. Evaporation of the immersion water was no longer insignificant due to the prolonged Raman imaging period. As a result, an automatic water immersion feeder employing syringe pumps and syringe needles affixed to the objective lens’s tip was used. Water was delivered at a flow rate of 1 μL/min.

### Raman Spectra Processing

Each raw Raman spectrum consists of 1340 channels. We extracted the fingerprint region covering 600–1800 cm^–1^ from these channels, yielding a revised set of 870 channels per spectrum. As a result, each FOV is represented as a 50 × 50 × 870 hyperspectral picture. A specific MATLAB (2020b) script was used to do the initial preprocessing of these hyperspectral pictures. Cosmic rays were detected by subtracting the median filtered spectra from the raw spectra, and any feature that differs from the median by more than three standard deviations is replaced with the median. This was accomplished using the hampel function in MATLAB. With a window size of 9 and a polynomial order of 5, Savitzky–Golay smoothing was used. Afterward, the baseline was subtracted with liberfit using a 12-order polynomial fit and 100 iterations. After the initial processing steps, the spectra were subjected to binning, specifically averaging 5 × 5 pixel regions, resulting in a 10 × 10 × 870 hyperspectral picture. As the last step, spectra were standardized using *z*-score, and normalization was performed using Frobenius.

### Data Analysis

The data set underwent dimensionality reduction using principal component analysis (PCA), where the number of components was determined based on the accuracy of the Random Forest (RF) algorithm. Specifically, we utilized the first 20 and 25 principal components to train the RF and employed *k*-fold cross-validation (*k* = 10) to validate the model. The data set was divided into 70% training and 30% testing sets. The RF classification was conducted by using an ensemble of 50 trees, serving as estimators in the ensemble. The identification of all pertinent predictors contributing to the classification task was determined by employing the Boruta algorithm with 10 iterations.

## Results

### Characterization of Maturation Stages of Pluripotent Stem Cell-Derived Cortical Organoids Using Raman Spectroscopy

**Figure 1:**
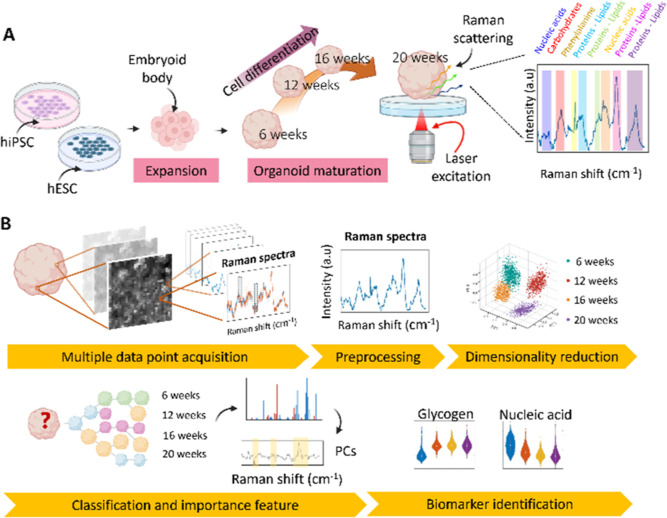
Overview. (A) Schematic of the organoid derivation, expansion, and maturation. Representation of the Raman measurement of the organoids at each maturation stage and the typical signal collected (Raman scattering) from the sample. (B) Experimental and analytical pipeline, encompassing data acquisition, preprocessing, multivariate analysis, ML, and biomarker identification.

The methodology proposed to discriminate between cortical organoid maturation stages involves the systematic recording of multiple organoids at different time points, specifically at 6, 12, 16, and 20 weeks (Figure 1A). During that time, the organoids increase in size and change their cellular composition—progressively become enriched in cortical neurons and decrease the number of neuronal progenitor cells.^16^ In particular, the selected time points capture key developmental milestones in cortical organoid maturation, including the transition to exclusively cortical cell populations after one month, peak neuronal diversity at two months, and the emergence of astroglia and immature interneurons between three and six months, as characterized by multiomics single-cell analyses and highlighted by Uzquiano et al.^16^ We measured three organoids per time point generated from the human-induced pluripotent cell line PGP1.

To facilitate RS measurements, the organoids were transferred to a phenol red-free Dulbecco’s modified Eagle’s medium (DMEM) in a quartz bottom Petri dish. The organoid was imaged using 785 nm excitation laser and Raman microscope developed in previous work by Kobayashi-Kirschvink et al.^17^ A schematic of this setup is depicted in Figure S1 of the Supporting Information.

The voxels of each map were binned 5 × 5 to reduce noise and enhance the signal-to-noise ratio. Subsequently, standard noise reduction techniques were applied, including baseline subtraction and cosmic rays removal (Figure 1B).

**Figure 2:**
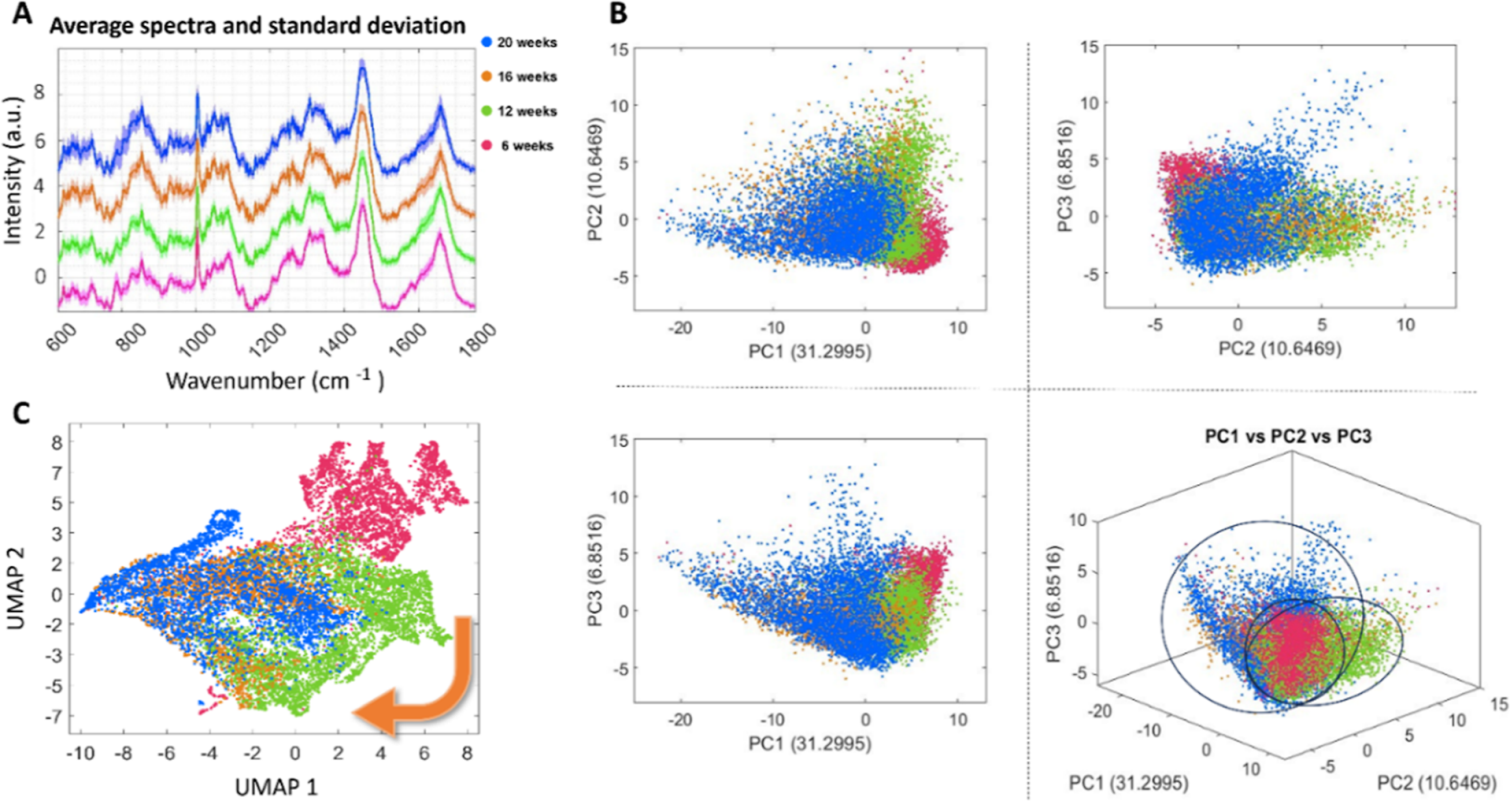
Raman spectra and multivariate analysis of PGP1-derived organoids. (A) Average Raman spectra calculated of the organoids at each development stage. (B) Score plot of the first three principal components and their explained variance (dots) colored by development stages. 3D plot of PC1, PC2, and PC3. (C) 2D UMAP embedding of binned Raman spectra (dots) colored by development stages.

In order to facilitate the comparisons among different organoids and maturation stages, data were further preprocessed using Frobenius normalization and *z*-score standardization.^32^ Frobenius normalization adjusts the overall variations in signal intensity, and the subsequent *z*-score normalization allowed the assessment of relative differences in spectral features within each maturation stage. The result of the preprocessing step is depicted in Figure 2a, where average spectra calculated for the different stages are plotted. Due to the high dimensionality of the Raman data set, PCA was employed to make the visual representation of the data more readable. The score plot (Figure 2B), which represents the projection of spectra onto principal components, shows slight variability within the four groups, indicating the stages of maturation of the cortical organoid. To facilitate data visualization further, we applied uniform manifold approximation and projection (UMAP), as shown in Figure 2C, since it enables visualization of high-dimensional data in a lower-dimensional space while preserving the intrinsic structure of the data. Through this visualization, it is possible to observe a developmental trajectory across the four stages, with clear transitions between earlier stages and more subtle differences emerging as development progresses, particularly between the 16 and 20 week time points. The data points are clustered according to their similarities and organized in a manner that reflects their developmental progression. This inherent directionality within the UMAP visualization provides an overall view of the transitions from one stage to the next.

**Figure 3:**
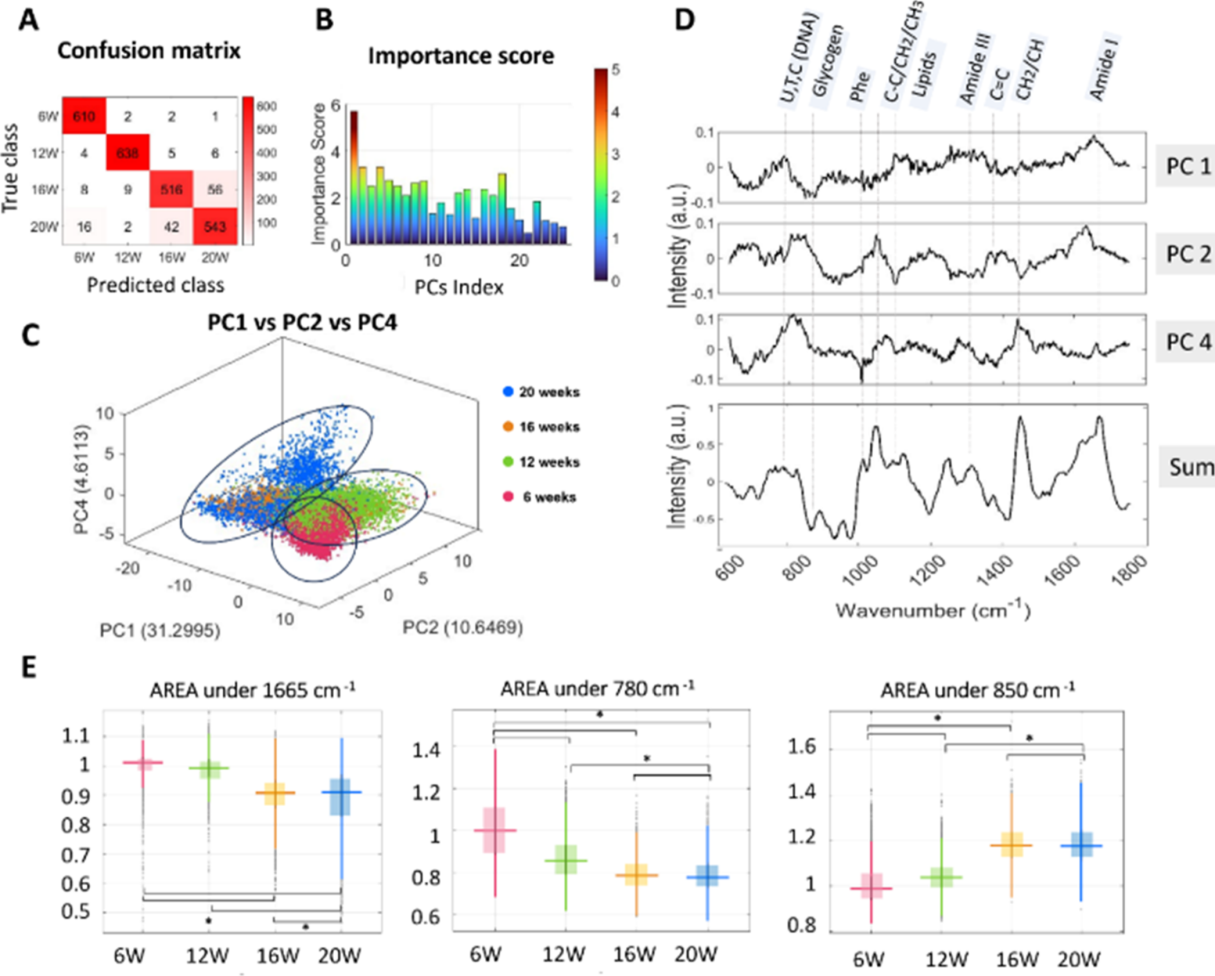
RF model for the classification of PGP1-derived organoid maturation stages and biomarker extraction. (A) Confusion matrix illustrating the performance of the classification model (B) Bar plot of the importance score extracted from RF across the first 25 principal components. (C) 3D score plot of the most important PCs, namely, PC1, PC2, and PC4. (D) Plot of the loadings as a function of Raman shift of the most important PCs and weighted sum of the first 25 PCs multiplied by importance coefficient. (E) Box plots of select peak intensities in the data set based on the ability to discriminate maturation stages (500 binned spectra collected from three independent organoids. Significance denoted by an asterisk (*) was determined at *p* < 0.05).

As a subsequent step, to extract feature importance from the multidimensional Raman spectra data set, we leveraged machine learning methodologies. Specifically, we employed the RF^28^ algorithm, a robust machine learning technique that harnesses the collective power of multiple decision trees. RF stands out for its transparency and interpretability, rendering it particularly well-suited for the analysis of complex, high-dimensional data sets like Raman spectra. Among the RF-based methods, the Boruta algorithm^28^ was employed to select essential predictor variables, thereby enhancing our understanding of the relationships between predictors and their impact on observed biochemical variations. The model was trained using the reduced data set employing the initial 25 principal components, which capture the most significant variability present in the data set. The data set was divided into a 70% training set and a 30% test set and to ensure robustness and validate the model’s performance; *k*-fold cross-validation with *k* set to 30 was employed. Remarkably, the model achieved an average accuracy score of 93.68% ± 0.54%, underscoring its proficiency in accurately predicting and classifying the data. To illustrate the performance of the model, we generated a confusion matrix comparing the predicted and the true value (Figure 3A), where the rows represent the true classes, while the columns represent the predicted classes. The diagonal elements indicate the number of correctly classified instances for each class, and the off-diagonal elements show misclassifications between classes. Moreover, we have included the complete classification metrics, including recall, precision, and F1-score, in Table S1 for a comprehensive evaluation of the model’s performance.

We observed that the model presents a higher error rate in classifying between the 16 week and 20 week stages. This discrepancy likely reflects the more subtle differences in the cortical organoids at later stages compared to the more pronounced dissimilarities that occur up to the third month due to heightened rates of cell differentiation and migration processes.^16^ By plotting feature importance across the first 25 principal components, we were able to estimate the contribution of each PC to the predictive performance of the RF (Figure 3B). PC1, 2, and 4 have the highest importance and therefore likely play a pivotal role in characterizing the data set. As a matter of fact, the use of PC1, PC2, and PC4 (Figure 3C) visualizes the difference between the 4 experimental groups better than if PC1, PC2, and PC3 are used (Figure 2B). Their loadings as a function of the Raman shift are plotted in Figure 3D, along with a tentative assignment of the most significant peaks. The assignment shows contribution of protein bands of amide I (1640–1680 cm^–1^), amide III (1220–1370 cm^–1^), and C–C stretching (928–940 cm^–1^) and phenylalanine (1004 cm^–1^) together with bands characteristic of lipids, CH2 scissoring vibration (1420–1450 cm^–1^), and CH2 twisting around 1300 cm^–1^. Moreover, possible changes in polysaccharide were found in the region 840–860 cm^–1^ (probably due to glycogen) ^33^and the ring breathing modes in the DNA/RNA bases bands (788 cm^–1^).^34^ To extract the cumulative differences between developmental stages, we calculated the weighted sum of the first 25 PCs, with each component multiplied by its respective coefficient of importance (feature importance) derived from the RF model. This represents a linear combination of the original principal components, emphasizing those that have a higher impact on the model’s predictions. In this way, we could highlight spectral regions most relevant to the classification task, identifying the primary contributors to the differences between the four maturation stages. The selected PCs collectively explain 61.4% of the total variance, ensuring that the weighted sum captures the majority of the system’s variability while prioritizing features most significant for distinguishing growth stages. We have observed that the loadings corresponding to nucleic acids (at 780 cm^–1^),^35^ glycogen (at 850 cm^–1^),^33^ and protein (at 1650 cm^–1^)^36^ best describe the changes live organoids undergo during the differentiation process (Figure 3D). We computed the area under these peaks within the original data set for the four maturation stages, revealing statistical differences between the groups at a significance level of 0.05 determined using the Mann–Whitney test (Figure 3E). Overall, throughout the course of organoid development in vitro, there was a progressive decrease in both nucleic acid and protein contents, while the amount of glycogen notably increased. These findings align with established developmental trajectory in cortical organoids, where metabolic shifts reflect the migration and differentiation of progenitor cells into mature neurons during cortical maturation.^16^ Furthermore, at 6 weeks, the distribution of nucleic acids exhibits a larger interquartile range, indicative of a greater standard deviation. This observation implies an increased degree of variability in the nucleic acid levels during this particular stage of development. This phenomenon can be attributed to the dynamic changes, migration, and layering of cells that occur during the maturation of brain organoids. Initially, at 1 month, clusters of apical radial glia progenitors occupy both central and peripheral regions of the organoid, along with intermediate progenitors and both mature and immature projection neurons.

However, by the third month, a loss of recognizable structure occurs, and a clearer layering of cell subpopulations emerges, with more similar cells locating toward the outermost layer, thereby increasing the homogeneity of this layer.^16^

To provide a possible minimum time interval achievable in detecting biological differences during cortical organoid maturation, we calculated the Euclidean distances between group centroids in the PCA space (Figure S2A,B). By focusing on PC1, PC2, and PC4, the components with the highest separability, we ensured that the distances reflect meaningful group differentiation based on the most significant patterns in the data. Using the smallest separable group (4–5 months, distance = 0.85) as a threshold, we extended this analysis to earlier developmental intervals. This approach allowed us to estimate the minimum detectable differences for groups between 6 and 12 weeks, resulting in 1 week, and between 12 and 16 weeks, 0.5 weeks. These results are shown in Figure S2C,D and emphasize the methodology’s capability to capture subtle, biologically relevant changes even within short timeframes.

### Validation of the Methodology: Raman Profiling of Embryonic Stem Cell-Derived Cortical Organoid Maturation Stages

**Figure 4:**
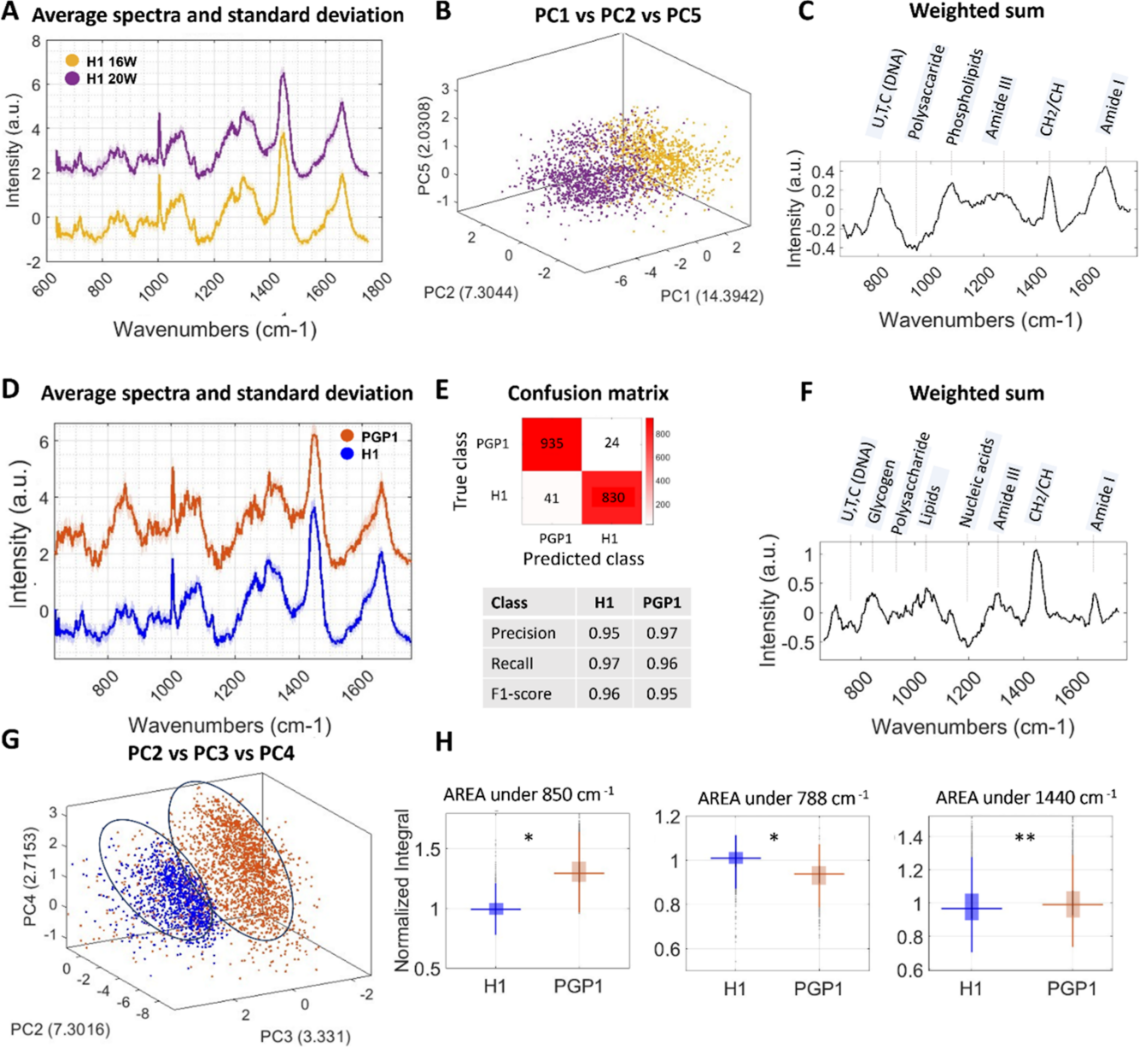
Raman spectra and classification of H1-derived organoid maturation stages and comparison with PGP1 Line. (A) Average Raman spectra calculated of the organoids at each development stage (16 weeks and 20 weeks) for H1-derived organoids. (B) 3D plot of the most important PCs (PC1, PC2, and PC5) extracted from the RF of the binned Raman spectra (dots) colored by development stages for H1-derived organoids. (C) Weighted sum of the importance coefficient multiplied by the respective PCs. (D) Average Raman spectra calculated of the organoids 16 and 20 weeks for H1-derived organoids and PGP1-derived organoids, respectively. (E) Confusion matrix, precision, recall, and F1-score of the two classes (H1-derived organoid and PGP1-derived organoids). (F) Weighted sum of the importance coefficient multiplied by the respective PCs. (G) 3D plot of the most important PCs (PC2, PC3, and PC4) extracted from the RF of the binned Raman spectra (dots) colored by cell line the organoids were derived from. (H) Box plot of select peak intensities in the data set based on the ability to discriminate maturation stages (significance denoted by an asterisk (*) at *p* < 0.05 and (**) at *p* < 0.01).

To verify the robustness of the methodology, organoids generated from a second male cell line were tested (H1; human embryonic stem cells). The goal was to assess the performance of the methodology on stages with more subtle differences, such as 16 and 20 weeks, to test its limits. Additionally, including different cell lines is crucial, as they may exhibit varied behaviors. In Figure 4A, we depict the average normalized Raman spectra obtained from H1-derived organoids at 16 and 20 weeks, computed following the preprocessing steps outlined in the previous section.

The data set comprises 2 organoids per maturation stage and a total of 8 FOV for each organoid. Subsequently, this data set underwent dimensionality reduction through PCA, followed by applying RF to the reduced data set. Remarkably, the algorithm discriminates between organoids at 16 and 20 weeks, with an accuracy of 0.96. The higher accuracy in this case is supposedly due to the fact that the algorithm is classifying between two different classes instead of four classes. For completeness, the confusion matrix and F1 score presented in Figure S3A of the Supporting Information demonstrate the algorithm’s classification performance, indicating good accuracy of the model. The bar plot in Figure S3B illustrates the feature importance, highlighting the most influential PCs in the classification process, namely, PC1, PC2, and PC5. Additionally, Figure S3C shows the loadings of these principal components (PC1, PC2, and PC5), revealing which Raman features contribute to the variance in the data and the overall classification accuracy. When PC1, PC2, and PC5 are used to plot the data, a clear separation of the two developmental stages of H1-derived organoids can be observed (Figure 4B). However, to avoid overlooking even small contributions and to identify the overall factors in this classification, the weighted sum of the 25 most important PCs is visualized in Figure 4C. To identify specific biomarkers that may contribute to this classification, we conducted a tentative assignment. This analysis revealed significant contributions from various molecular bands, including protein bands in amide I (1640–1680 cm^–1^) and amide III (1220–1370 cm^–1^),^36^ lipid in CH2 scissoring vibration (1420–1450 cm^–1^), polysaccharides in the region 940–960 cm^–1^, and ring breathing modes in the DNA/RNA bases bands (788 cm^–1^).^34^ From this analysis we extract the contribution of specific biomarkers, and the trends in these biomarkers are presented in Figure S3D. Specifically, a reduction in protein levels at 1350 cm^–1^ and nucleic acids, as indicated by the peak at 788 cm^–1^, is observed, alongside an increase in the polysaccharide content at 940 cm^–1^ with a significance level of 0.05. Remarkably, the proposed analytical method demonstrated the efficacy of RS in distinguishing between the 16 week and 20 week maturation stages in H1-derived cortical organoids, as was shown previously for PGP1-derived organoids.

### Comparative Analysis between Cortical Organoids from Embryonic and Pluripotent Stem Cells Using Raman Spectroscopy

The use of different cell lines for directed differentiation can moderately affect the resulting biological readout; therefore, it is an ideal model for testing the robustness of our methodology. To this end, approximately 40 FOVs were analyzed in each of four organoids generated from either H1 or PGP1 (two organoids at 16 weeks and two at 20 weeks).^37^ We computed the average Raman spectra for PGP1- and H1-derived organoids at both 16 and 20 weeks, as shown in Figure 4D. In the figure, the brown line represents the average spectra for PGP1-derived organoids between 16 and 20 weeks, while the blue line represents the average for H1-derived organoids. The resulting Raman spectra displayed discernible variations between the two cell lines, as depicted in Figure 4D, offering valuable insights into their respective biochemical characteristics.

Following preprocessing and dimensionality reduction, we employed the RF algorithm to extract the most important features. Specifically, we trained the algorithm using PCs 2 through 20. Notably, the decision to exclude the first principal component (PC1), despite encapsulating the highest variability within the sample (with an explained variance of 42.7%), was motivated by its lack of interpretability and relevance to the underlying patterns in the data. Since PC1 does not align with any known patterns or provide valuable biochemical insights, it appears to predominantly represent noise rather than meaningful features, potentially influenced by the substrate. Therefore, excluding PC1 ensures that subsequent analyses focus on more relevant and interpretable components, enhancing the quality and reliability of the results. By leveraging the 20 most significant PCs, we achieved an average accuracy of 0.97, compared to 0.96 when PC1 was included in the analysis. The confusion matrix along with metrics such as recall, precision, and F1-score in Figure 4E provides a comprehensive evaluation of the model’s performance. These metrics offer insights into the model’s ability to correctly classify samples across different cell lines, crucial for assessing its overall effectiveness and reliability in the classification between H1 organoids and PGP1 organoids. Additionally, Figure 4F showcases the weighted sum of the loadings of the first 20 most important components, multiplied by their relative importance as calculated through the RF algorithm, providing an indication into the contributions of these components to the classification process. These loadings indicate that there is high importance in the weight of the protein bands in amide I (1640–1680 cm^–1^) and amide III (1220–1370 cm^–1^),^36^ for the lipid in CH2 scissoring vibration (1420–1450 cm^–1^), lipids, and phospholipids (1080 cm^–1^). We can also observe changes in polysaccharides in the region 940–960 cm^–1^ and in the region 840–860 cm^–1^ (glycogen)^33^ and contribution on the nucleic acids in 1200 cm^–1^ and the ring breathing modes in the DNA/RNA bases bands (788 cm^–1^).^34^ Notably, the most prominent difference is attributed to glycogen levels at 850 cm^–1^,^33^ lipids level (1440 cm^–1^), and nucleic acid level (788 cm^–1^)^35^ (Figure 4F). Specifically, the PGP1 cell line exhibits a higher value in glycogen and in lipid concentration, together with a decreasing level in nucleic acids compared to the H1-derived organoid.

## Discussion

We introduced an innovative strategy for classifying the maturation stages of living human-derived cortical organoids using RS, with a focus on capturing Raman spectral signatures from the external outer layers. Our method integrates confocal RS with the RF classification algorithm, showing exceptional accuracy in categorizing organoid stages. This algorithm not only enables precise classification but also facilitates the extraction of feature importance, allowing for the identification of relevant biomarkers. Moving away from conventional multivariate analysis, we have shifted our focus to emphasizing the features that predominantly contribute to variability in the system. By training the RF algorithm to assign importance to each PC in the classification process, we gain insight into which components carry the most weight in discriminating between maturation stages and across different cell lines. Then, utilizing a linear combination of the original PCs, represented as a weighted sum, provides an overall indication of biochemical variation without prior bias toward specific PCs. This approach offers comprehensive insight into the classification process by considering the collective contributions of all PCs rather than focusing on individual components.

To validate our approach, we applied it to two distinct organoid lines derived from induced pluripotent stem cells (PGP1) and embryonic stem cells (H1). We observed clear differences between the maturation stages in both cases, albeit with slightly different biochemical profiles. Specifically, from 6 to 20 weeks in PGP1-derived organoids, we observed an increase in protein and glycogen levels alongside a decrease in nucleic acids. Conversely, in H1-derived organoids, we observed an overall increase in polysaccharides and a decrease in nucleic acid and protein levels between 16 and 20 weeks. Therefore, these biomarkers could serve as indicators for the proper maturation of human cortical organoids, rendering this analysis a label-free methodology for assessing maturation quality.

Furthermore, we compared the Raman signatures from the two cell lines and extracted differences using the same approach. The analysis revealed notable differences, particularly in glycogen, lipids, and nucleic acids, with significantly higher levels of glycogen observed in the PGP1 cell line. This discrepancy suggests potential variations in the metabolic activity or storage mechanisms between the two cell lines. Higher glycogen levels in the PGP1 cell line could indicate increased energy storage capacity or utilization compared to that in the H1 cell line. Additionally, differences in lipid and nucleic acid levels may reflect variations in cellular phenotypes. Understanding these biochemical distinctions is invaluable for longitudinal studies involving multiple cell lines, offering deeper insights into their unique physiological characteristics and functional behaviors.

## Conclusions

In conclusion, our study highlights the feasibility of utilizing RS to monitor the maturation stages of cortical organoids. We demonstrate the potential of RS, when coupled with machine learning algorithms, to accurately discriminate between these stages and extract valuable biochemical information in a noninvasive and label-free manner. Specifically, the ability to measure organoid maturation in a label-free, noninvasive manner has transformative potential for various sectors, including regenerative medicine, where it can guide tissue engineering and transplantation strategies; drug discovery and development, by enabling high-throughput screening and real-time assessment of drug efficacy and toxicity; and disease modeling, through longitudinal studies that replicate human developmental and pathological processes in vitro, ultimately advancing personalized medicine and therapeutic innovation.

Moreover, in the future, integrating label-free RS with established methods such as electrical recording,^38^ other noninvasive imaging techniques,^39^ or multiomics will offer significant advantages, particularly in assessing drug efficacy and neurodevelopment. Furthermore, RS can be integrated with microfluidics^40^ and intracellular recording,^41,42^ specifically to increase the specificity and sensitivity of measurements. By harnessing the unique capabilities of RS, researchers can further explore cellular responses and acquire valuable information that complements and enriches the findings obtained through conventional techniques in both pathological and physiological studies.

## Supporting Information Available

Schematic of the multimodal Raman microscope, summary of classification metrics for RF model performance, minimum detectable time intervals during PGP1 cortical organoid maturation, and RF model for classification of H1-derived organoid maturation stages (PDF)
