# Supplementary material for: Label-Free Detection of Biochemical Changes during Cortical Organoid Maturation via Raman Spectroscopy and Machine Learning
Source: Anal Chem. 2025 Feb 24;97(9):5029–37. doi: 10.1021/acs.analchem.4c05661 (PMC11912127; doi:10.1021/acs.analchem.4c05661)
Supplement: Supplementary file 1 — ac4c05661_si_001.pdf [file ac4c05661_si_001.pdf]

Supporting Information

Title

Label-Free detection of biochemical changes during cortical organoids maturation via Raman spectroscopy and Machine Learning

Authors

Giulia Bruno * [1,2], Michal Lipinski[3], Koseki J. Kobayashi-Kirschvink[2,3], Christian Tentellino[1], Peter T. C. So[2], Jeon Woong Kang[2], Francesco De Angelis[1]

[1] Istituto Italiano di Tecnologia, via Morego 30, 16163, Genova, Italy

[2] G. R. Harrison Spectroscopy Laboratory, Massachusetts Institute of Technology, Cambridge, MA, 02139, USA

[3] Broad Institute of MIT and Harvard, 415 Main St, Cambridge, MA 02142, USA

Table of Contents

Figure S1 A schematic of the multimodal Raman microscope **2**
Table S1 Summary of classification metrics for Random Forest model performance **3**
Figure S2 Minimum detectable time intervals during PGP1- cortical organoid maturation **4**
Figure S3 Random Forest model for classification of H1 derived organoid maturation stages and biomarkers extraction H1-derived organoid. **5**

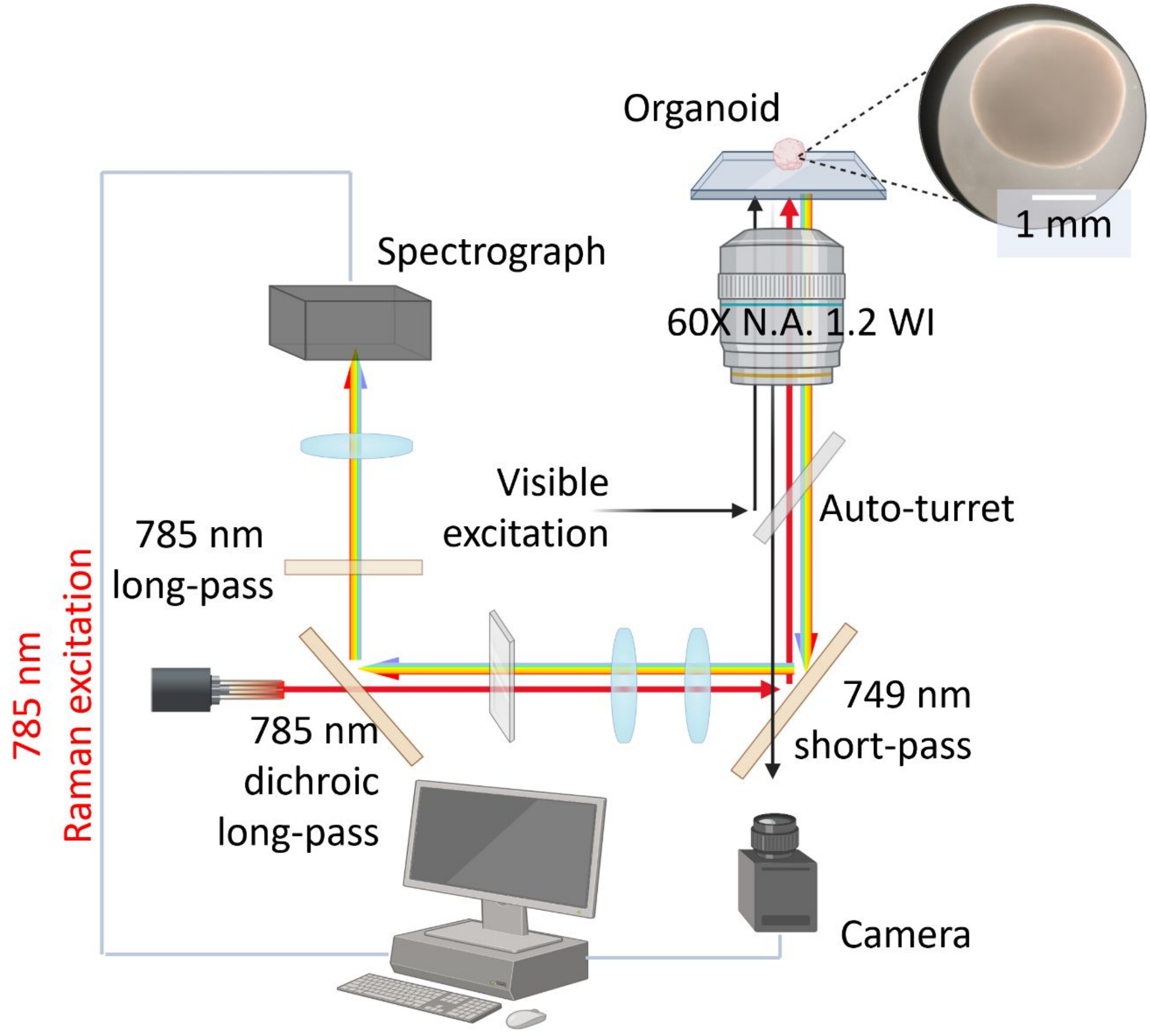

Figure S1 A schematic of the multimodal Raman microscope

Class	Precision	Recall	F1-score
6 W	99.78 ± 0.3	98.3 ± 0.7	99.0 ± 0.4
12 W	97.87 ± 0.7	97.3 ± 0.9	97.6 ± 0.5
16 W	87 ± 1	93 ± 1	90.1 ± 0.8
20 W	93 ± 1	89 ± 1	91 ± 1

Table S2 Summary of classification metrics for Random Forest model performance. The table includes recall, precision, F1-score, and accuracy values for each class, along with their standard deviations across 30 cross-validation trials

A

Euclidean distance

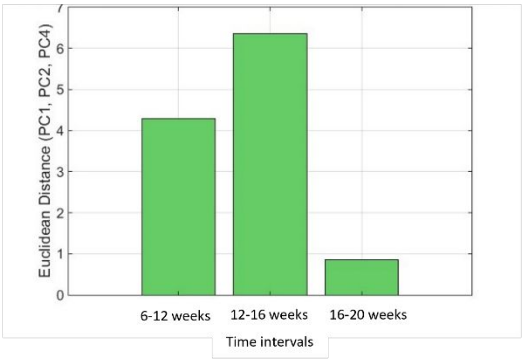

B

Group 1	Group 2	Euclidean distance
6 weeks	12 weeks	4.29
6 weeks	16 weeks	8.88
6 weeks	20 weeks	9.70
12 weeks	16 weeks	6.35
12 weeks	20 weeks	7.02
16 weeks	20 weeks	0.85

C

Minimum detectable distance (first interval)

D

Minimum detectable distance (second interval)

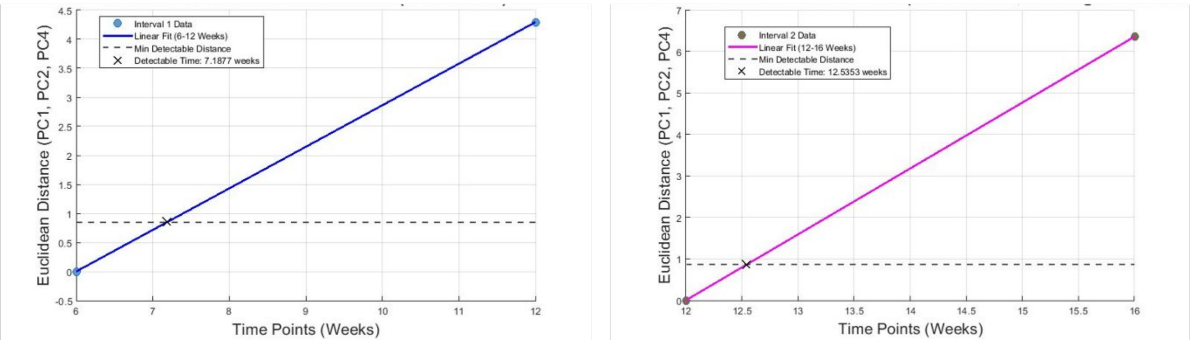

Figure S2 Minimum detectable time intervals during PGP1- cortical organoid maturation. (A) Pairwise comparison of Euclidean distances between developmental stages, with the smallest separable group (16-20 months) identified at a threshold of 0.85. (B) Euclidean distances between group centroids in the PCA space for PC1, PC2, and PC4, highlighting separability between maturation stages (6, 12, 16, and 20 weeks). (C-D) Estimated minimum detectable differences for earlier intervals. Groups between 6 and 12 weeks correspond to a minimum detectable interval of 1 week, and between 12 and 16 weeks, 0.5 weeks.

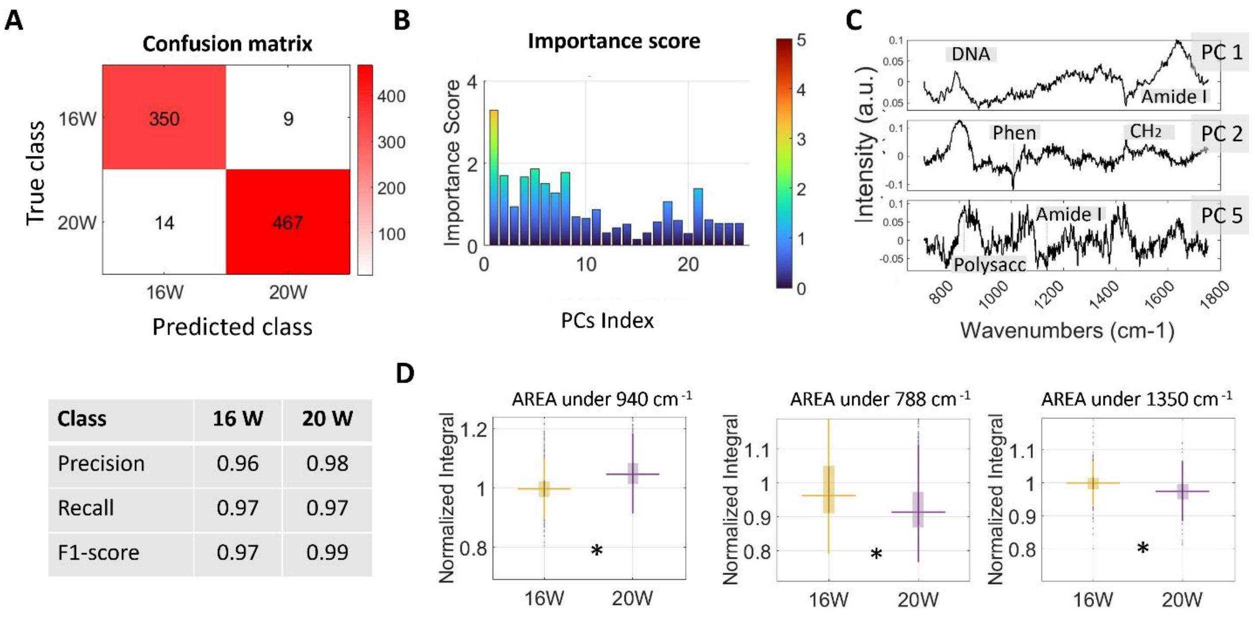

Class	16 W	20 W
Precision	0.96	0.98
Recall	0.97	0.97
F1-score	0.97	0.99

Figure S3 Random Forest model for classification of H1 derived organoid maturation stages and biomarkers extraction H1-derived organoid. (A) Confusion martrix and a table showing Precision,Recall and F1-score of the predicted class illustrating the performance of the model (B) Bar plot of the PCs coefficient importance extracted from RF across the first 25 principal components. (C) Plot of the loadings as function of Raman shift of the most important PCs, namely PC1, PC2 and PC5. (D) Box plots of select peak intensities in the dataset based on the ability to discriminate maturation stages (500 binned spectra collected from three independent organoids. Significance denoted by an asterisk (*), was determined at $p < 0.05$).
